# Supplementary material for: Comparative multiomics analysis of cell physiological state after culture in a basket bioreactor
Source: Sci Rep. 2022 Nov 23;12:20161. doi: 10.1038/s41598-022-24687-4 (PMC9686226; doi:10.1038/s41598-022-24687-4)
Supplement: Supplementary file 1 — Supplementary Information 1. [file 41598_2022_24687_MOESM1_ESM.zip › raw data/Transcriptomic raw data/6.Enrichment/2.KEGG/TE1vsTC1/ALL/src/csab04151.html]

csab04151


- csab:103224900

- Down regulated genes

103224900(-1.42998371610984)

- csab:103225530

- Up regulated genes

103225530(1.52855282236579)
- csab:103246479

- Down regulated genes

103246479(-1.5490263767958)

- csab:103237430

- Up regulated genes

103237430(1.35562492669487)

- csab:103246489

- Up regulated genes

103246489(1.48197213715262)

- csab:103235805

- Up regulated genes

103235805(2.54406016949455)
- csab:103235804

- Up regulated genes

103235804(2.53749812915487)

- csab:103224900

- Down regulated genes

103224900(-1.42998371610984)

- csab:103243277

- Up regulated genes

103243277(1.88743299439371)

- csab:103214795

- Up regulated genes

103214795(1.23734090959605)

- csab:103223920

- Down regulated genes

103223920(-1.66241974207187)

- csab:103226390

- Up regulated genes

103226390(2.68260263702081)
- csab:103237005

- Down regulated genes

103237005(-1.92426444229972)

- csab:103247197

- Up regulated genes

103247197(1.34087353024886)

- csab:103245773

- Down regulated genes

103245773(-2.38331667842751)

- csab:103246489

- Up regulated genes

103246489(1.48197213715262)

- csab:103238335

- Up regulated genes

103238335(2.31055276582108)

Close
